# Identification of novel *TMEM231* gene splice variants and pathological findings in a fetus with Meckel Syndrome

**DOI:** 10.3389/fgene.2023.1252873

**Published:** 2023-09-06

**Authors:** Qian Zhang, Shuya Yang, Xin Chen, Hongdan Wang, Keyan Li, Chaonan Zhang, Shixiu Liao, Litao Qin, Qiaofang Hou

**Affiliations:** ^1^ Henan Provincial Key Laboratory of Genetic Diseases and Functional Genomics, Henan Provincial People’s Hospital, Medical Genetics Institute of Henan Province, People’s Hospital of Zhengzhou University, Zhengzhou University, Zhengzhou, China; ^2^ National Health Commission Key Laboratory of Birth Defects Prevention, Henan Key Laboratory of Population Defects Prevention, Zhengzhou, China; ^3^ People’s Hospital of Henan University, Henan University, Zhengzhou, China

**Keywords:** Meckel Syndrome, *TMEM231* gene, whole exome sequencing, splicing mutation, alternative transcription, primary cilia

## Abstract

**Background:** Meckel Syndrome (MKS, OMIM #249000) is a rare and fatal autosomal recessive ciliopathy with high clinical and genetic heterogeneity. MKS shows complex allelism with other related ciliopathies such as Joubert Syndrome (JBTS, OMIM #213300). In MKS, the formation and function of the primary cilium is defective, resulting in a multisystem disorder including occipital encephalocele, polycystic kidneys, postaxial polydactyly, liver fibrosis, central nervous system malformations and genital anomalies. This study aimed to analyze the genotype of MKS patients and investigate the correlation between genotype and phenotype.

**Methods:** A nonconsanguineous couple who conceived four times with a fetus affected by multiorgan dysfunction and intrauterine fetal death was studied. Whole exome sequencing (WES) was performed in the proband to identify the potentially pathogenic variant. Sanger sequencing was performed in family members. In silico tools were used to analyse the pathogenicity of the identified variants. cDNA TA-cloning sequencing was performed to validate the effects of intronic variants on mRNA splicing. Quantitative real-time PCR was performed to investigate the effect of the variants on gene expression. Immunofluorescence was performed to observe pathological changes of the primary cilium in kidney tissue from the proband.

**Results:** Two splice site variants of *TMEM231* (NM_001077418.2, c.583-1G>C and c.583-2_588delinsTCCTCCC) were identified in the proband, and the two variants have not been previously reported. The parents were confirmed as carriers. The two variants were predicted to be pathogenic by *in silico* tools and were classified as pathogenic/likely pathogenic variants according to the American College of Medical Genetics and Genomics guideline. cDNA TA cloning analysis showed that both splice site variants caused a deletion of exon 5. RT-PCR revealed that the expression of *TMEM231* was significantly decreased and immunofluorescence showed that the primary cilium was almost absent in the proband’s kidney tissue.

**Conclusion:** We reported the clinical, genetic, molecular and histochemical characterisation of a family affected by MKS. Our findings not only extended the mutation spectrum of the *TMEM231* gene, but also revealed for the first time the pathological aetiology of primary cilia in humans and provide a basis for genetic counselling of the parents to their offspring.

## Introduction

Meckel Syndrome (MKS, OMIM #249000) is a rare and fatal autosomal recessive ciliopathy with an incidence ranging from 1:13,250 to 1:140,000 (Young et al., 1985; Yaqoubi and Fatema, 2018; Lin et al., 2022). MKS is a highly clinically heterogeneous and multisystem disorder characterised by classic features—occipital encephalocele, polycystic kidneys, postaxial polydactyly and other anomalies (including liver fibrosis, central nervous system malformations and genital anomalies) (Logan et al., 2011; Hartill et al., 2017; Liu et al., 2021). The aetiology of MKS is the dysfunction of the primary cilium during early embryogenesis, and mutations in ciliary-associated proteins result in a group of disorders including Meckel Syndrome (MKS, OMIM #249000), Polycystic kidney disease (PKD, OMIM #173900), nephronophthisis (NPHP, OMIM #256100), Bardet-Biedl Syndrome (BBS, OMIM #209900), Joubert syndrome (JBTS, OMIM #213300) and Alstrom syndrome (ALMS, OMIM #203800) (Badano et al., 2006; Sharma et al., 2008; Yuan and Sun, 2013; McConnachie et al., 2021; Moreno-Leon et al., 2022).

Seventeen genes are found to be linked with MKS, and the *MKS1* gene mutations cause 16% of MKS cases (Iannicelli et al., 2010; Shaheen et al., 2013; Wang et al., 2021). The *TMEM231* gene, identified in 2013 as a causative gene for MKS, also contributes to JBTS which is another ciliopathy characterised by oculomotor apraxia, abnormal breathing, ataxia, and variable psychomotor developmental delay (Srour et al., 2012; Shaheen et al., 2013; Wang et al., 2021). MKS and JBTS exhibit common clinical features such as polydactyly and renal involvement. A clear clinical diagnosis for MKS is often delayed due to its low frequency and complex phenotype.

With the development of sequencing technologies and the reduction of technology costs, whole-exome sequencing (WES) has been widely applied to reveal the genetic aetiology of congenital diseases, especially in rare diseases with extreme genotypic and phenotypic heterogeneity (Chen et al., 2021; Pan et al., 2022; Wang et al., 2022; Zhang et al., 2022). Here, we collected a pedigree of four siblings affected by MKS and identified two novel splice site variations of the *TMEM231* gene (NM_001077418.2; c.583-1G>C and c.583-2_588delinsTCCTCCC) by WES. We used alternative splicing experiments, mRNA expression detection and immunofluorescence techniques to investigate the correlation between genotypes and phenotypes.

## Materials and methods

### Participants

**FIGURE 1:**
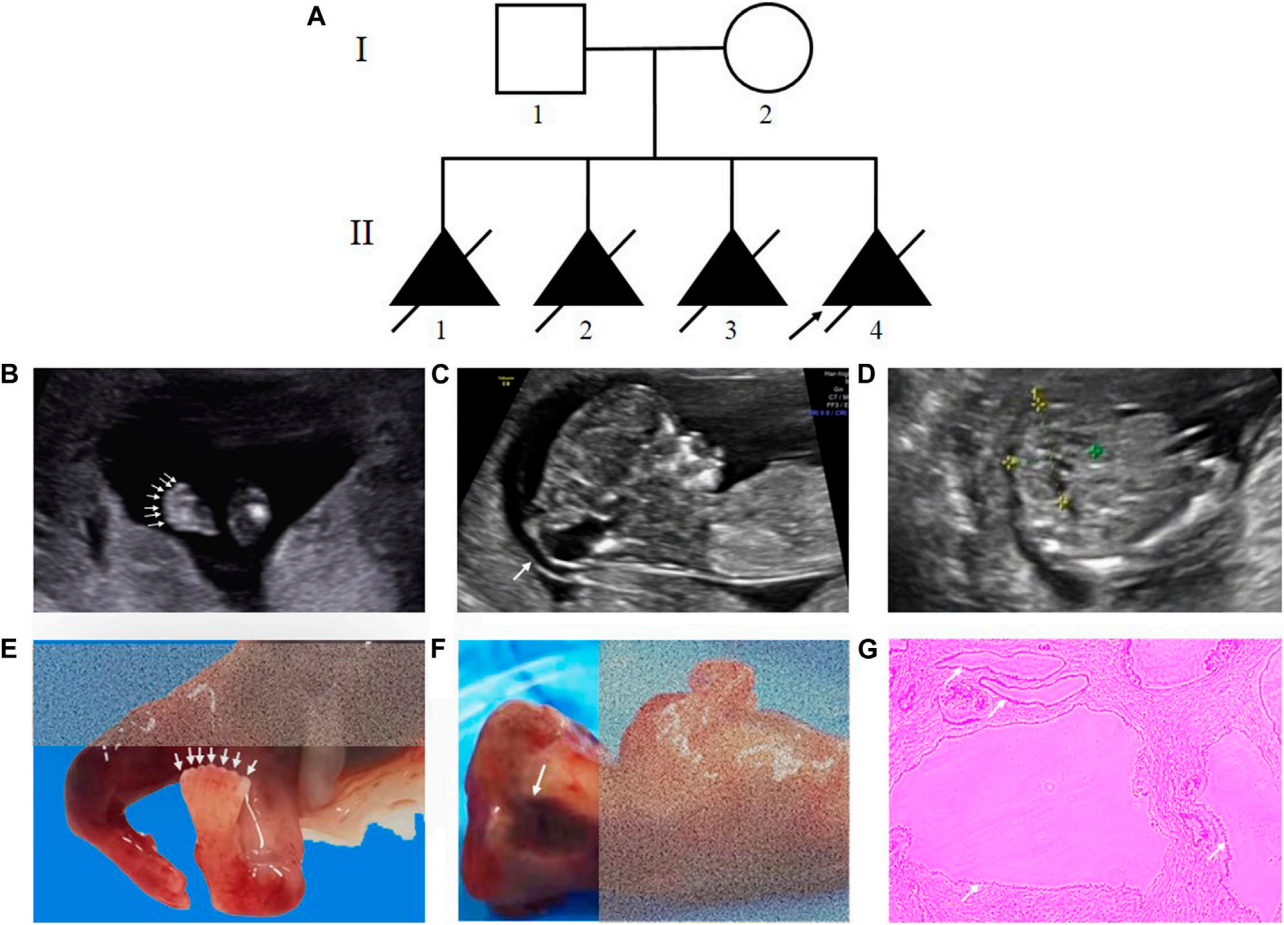
Pedigree of the family and clinical features of the fetus affected by MKS. **(A)** Pedigree of the family. The affected fetuses are marked with solid triangles. **(B)**, **(C)** and **(D)** Ultrasound examination showed postaxial polydactyly of the feet, occipital encephalocele and bilateral polycystic kidneys. **(E)**, **(F)** Necropsy showed the proband with seven toes of the feet and anencephaly. **(G)** HE staining of kidney tissue from the proband.

The nonconsanguineous couple came for genetic counselling at the Medical Genetics Institute of the Henan Provincial People’s Hospital. At 13 weeks, ultrasound revealed that the couple’s fourth fetus had intrauterine fetal death and multiorgan dysfunction. Investigation of their family history unveiled that the couple had similar reproductive history on three previous occasions. Figure 1A displays the family pedigree. Clinical investigations, including an ultrasound scan, were carried out in the pertinent clinical departments. The Ethics Committee of the Henan Provincial People’s Hospital approved this study (No. 2019-134), and informed consent was obtained from the couple.

### Whole-exome sequencing and data analysis

The genomic DNA (gDNA) was extracted from the peripheral blood of the couple and the fetal skin utilizing the Qiagen genomic DNA isolation kit (Qiagen, Hilden, Germany). WES analysis was carried out on the gDNA of the proband (Ⅱ-4).

The Agilent SureSelect Human ALL Exon Kit v6 (Agilent Technologies, CA, United States) was used following the manufacturer’s protocol to generate the WES library. The prepared library was sequenced on the Illumina Nova sequencing platform (Illumina, CA, United States) with an average coverage depth of 100 × of the targeted regions, including coding regions and exon-intron boundaries. Alignment of clean reads to the human reference genome (GRCh37) was done using the BWA program and GATK software. Variants were filtered based on their minor allele frequencies (MAF) < 1% in the dbSNP (https://www.ncbi.nlm.nih.gov/snp), 1000 Genomes Project (1000 GP, https://browser.1000genomes.org) and Exome Variant Server (EVS, https://evs.gs.washington.edu/EVS) databases. The Human Gene Mutation Database (HGMD, https://www.hgmd.cf.ac.uk/ac/index.php), ClinVar (https://www.ncbi.nlm.nih.gov/clinvar/). GeneSplicer (http://www.cbcb.umd.edu/software/GeneSplicer/gen_spl.shtml) and Human Splicing Finder (http://www.umd.be/HSF) online software were used to predict and analyse the function of suspected splicing variants. All suspected variants were categorised according to the American College of Medical Genetics and Genomics (ACMG) guideline.

### Sanger sequencing

Sanger sequencing was conducted in the proband and family members to further authenticate the identified variants detected by WES. The primers used for Sanger sequencing are listed in Table 1 (Primer #1).

**TABLE 1:** Primers used in this study.

Name	Primers
Primer #1	*TMEM231*-F-5′-*CCGCTGAGATAAAGAGGGGT* -3′
	*TMEM231*-R-5′-*AAGACAAATCCTGGACAGCAT*-3′
Primer #2	*TMEM231*-mrE3-F-5′-*CGTTTCTCCAGTCCTCCTTTC*-3′
	*TMEM231*-mrE5-R:5′-*GCTATGGGACACCTTCAGTAAA*-3′
Primer #3	M13F-*TGTAAAACGACGGCCAGT*
	M13R-*CAGGAAACAGCTATGACC*
Primer #4	*TMEM231*-mr*E3-F:5′- CCC​GGG​ATC​CCA​GTT​ATA​CG-3′*
	*TMEM231-*mr*E5-R: 5′- TTG​GGA​TCA​TTC​AGG​ACG​GTG-3′*
Primer #5	*β-actin-F-5′- CCT​TCC​TGG​GCA​TGG​AGT​C-3′*
	*β-actin-R-5′- TGA​TCT​TCA​TTG​TGC​TGG​GTG-3′*

### Total RNA extraction and reverse-transcription

Total RNA was extracted from the whole blood of the couple and different organs from the fetal autopsy body utilizing the TRIzol reagent (Life Technologies, MA, United States). Total RNA was reverse transcribed to cDNA using the Revert Aid First Strand cDNA Synthesis Kit (Thermo Scientific, MA, United States).

### cDNA TA-cloning sequencing

To investigate the function of two putative splice-site mutations, TA cloning sequencing was performed on cDNA from the couple (I-1 and I-2). The PCR products were purified and cloned into pClone007 vector (TsingKe Biotechnology, Beijing, China). The recombinant vectors were transformed into TreliefTM5α-competent cells (TsingKe Biotechnology, Beijing, China) and ten monoclonal colonies were sequenced. The primers are listed in Table 1 (Primer #2 and Primer #3).

### Quantitative real-time PCR

Quantitative real-time PCR (qPCR) was performed to quantify the mRNA expression of *TMEM231* in the heart, kidney, and liver of the proband (Ⅱ-4). qPCR was conducted with SYBR Green qPCR Master Mix (Thermo Fisher, CA, United States) on an Applied Biosystems StepOne Real-Time PCR System (Thermo Scientific, MA, United States). The datasets were processed according to the 2^−ΔΔCT^ method and normalised it to the housekeeping gene *β-actin*. The primers are listed in Table 1 (Primer #4 and Primer #5).

### Data analysis

The data were analysed employing IBM SPSS Statistics 13.0 software. The within-sample analyses were conducted by Student's t-tests. The outcomes are demonstrated as mean ± SEM. A *p*-value of <0.05 was deemed statistically significant.

### Immunofluorescence

Kidney tissues from the proband (Ⅱ-4) were fixed in 10% paraformaldehyde and immunofluorescence was performed as a routine protocol. The primary antibody was ARL13B (Proteintech, 1711-1-AP, rabbit, 1:250), a specific marker for primary cilia, and the secondary antibody was anti-rabbit Alexa Fluor 488 (Abcam, ab150077, goat, 1:250). Immunofluorescence images were captured using a scanning microscope system (Nikon, Tokyo, Japan).

## Results

### Clinical presentation and family history

The proband (Ⅱ-4) was a fetus conceived naturally, and its ultrasound scan at 13 weeks revealed several anomalies, mainly ①occipital encephalocele (9.6 mm × 7.9 mm), ②bilateral polycystic kidneys, ③postaxial polydactyly of the hands and feet (Figures 1B–D, Supplementary Table S1). The necropsy results of the proband were consistent with the ultrasound findings, revealing anencephaly, bilateral enlarged polycystic kidneys, six fingers of the hands, and seven toes of the feet (Figures 1E–G). The histological testing showed thin cortex and glomerular atrophy in the proband’s kidneys with no abnormalities in the proband’s liver. The third fetus of the couple had similar ultrasound features at 17 weeks, including an occipital encephalocele (23 mm × 15 mm), bilateral enlarged polycystic kidneys, and bilateral polydactyly (Supplementary Figure S1). A summary of the medical history and clinical features of the four affected siblings is given in Table 2.

**TABLE 2:** Clinical information of the four siblings.

Sibling	Occipital encephalocele	Polycystic kidneys	Polydactyly	Bilateral talipes equinovarus	Pregnancy outcomes	Diagnostic time
Ⅱ-1	**+**	**-**	**-**	**-**	Termination	18 Weeks
Ⅱ-2	**+**	**+**	**-**	**-**	Termination	17 Weeks
Ⅱ-3	**+**	**+**	**+**	**-**	Termination	17 Weeks
Ⅱ-4	**+**	**+**	**+**	**+**	Termination	13 Weeks

Ⅱ-4 was the proband. Plus sign (+) indicates a positive characteristics. Minus sign (−) indicates a negative characteristic.

### Identified two novel mutations in the *TMEM231* gene by WES and sanger sequencing

To determine the genetic aetiology of this family, WES was performed and two novel heterozygous splice site mutations of the *TMEM231* gene (NM_001077418.2, c.583-1G>C and c.583-2_588delinsTCCTCCC) were detected in the proband (Ⅱ-4). These two variants were not recorded in the dbSNP, 1000 GP, EVS, ClinVar and HGMD databases. The online software GeneSplicer and Splicing Finder predicted that these two variants would lead to alternative splicing.

**FIGURE 2:**
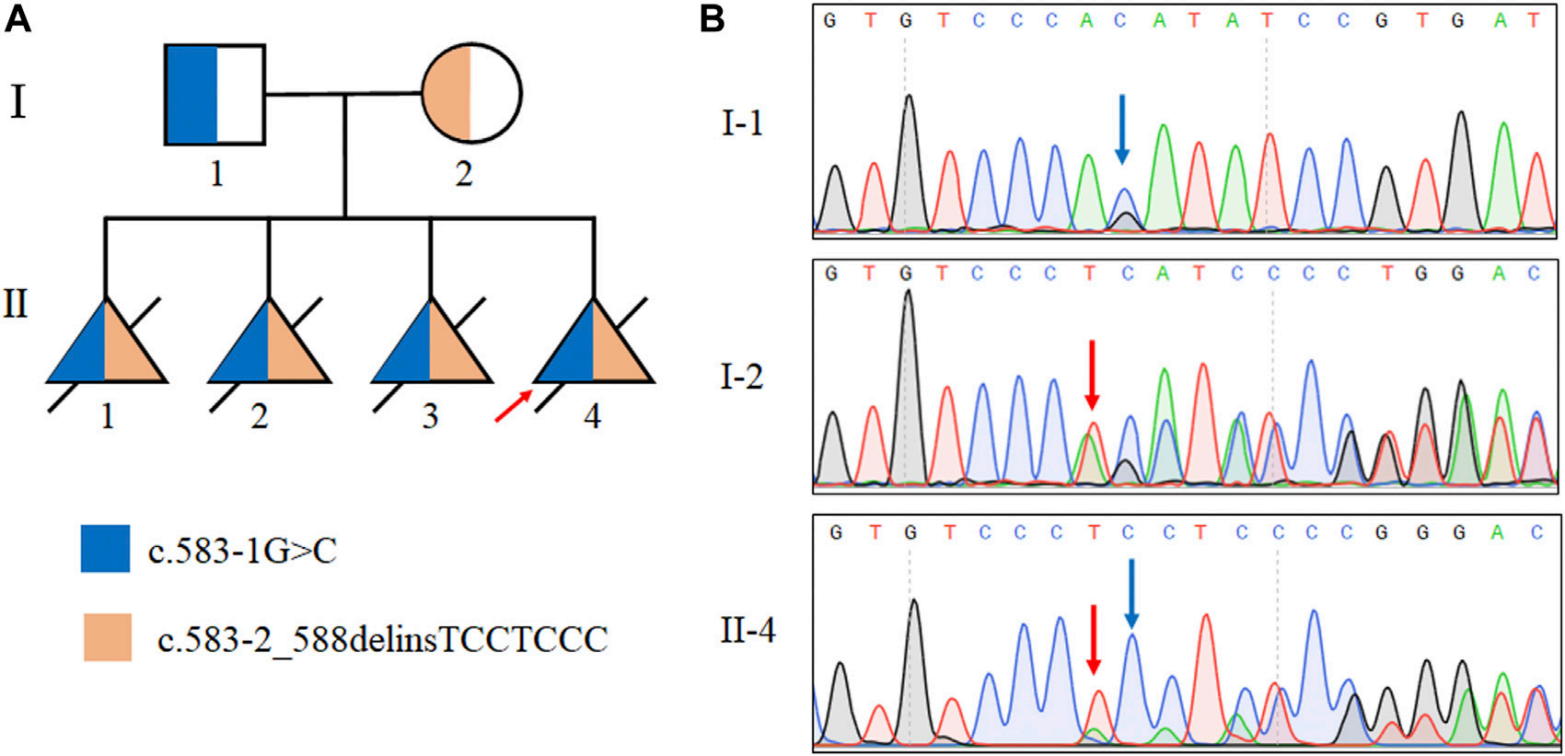
Identification of mutations in the *TMEM231* gene. **(A)** Pedigree with autosomal recessive MKS of two splice site mutations in this family. Ⅱ:1, Ⅱ:2 and Ⅱ:3 were assumed to be compound heterozygotes of the two splice site mutations. **(B)** Sanger sequencing results of the *TMEM231* gene variants of the proband (Ⅱ-4), the proband’s father (I-1) and the proband’s mother (I-2).

Through Sanger sequencing we confirmed that the two mutations were inherited from the proband’s father and mother, respectively (Figure 2). Overall, both classical splice site variants were classified as pathogenic according to the ACMG guidelines (PVS1+PM2+PP4).

### Two splice-site mutations caused the same pathogenic alternative transcription

**FIGURE 3:**
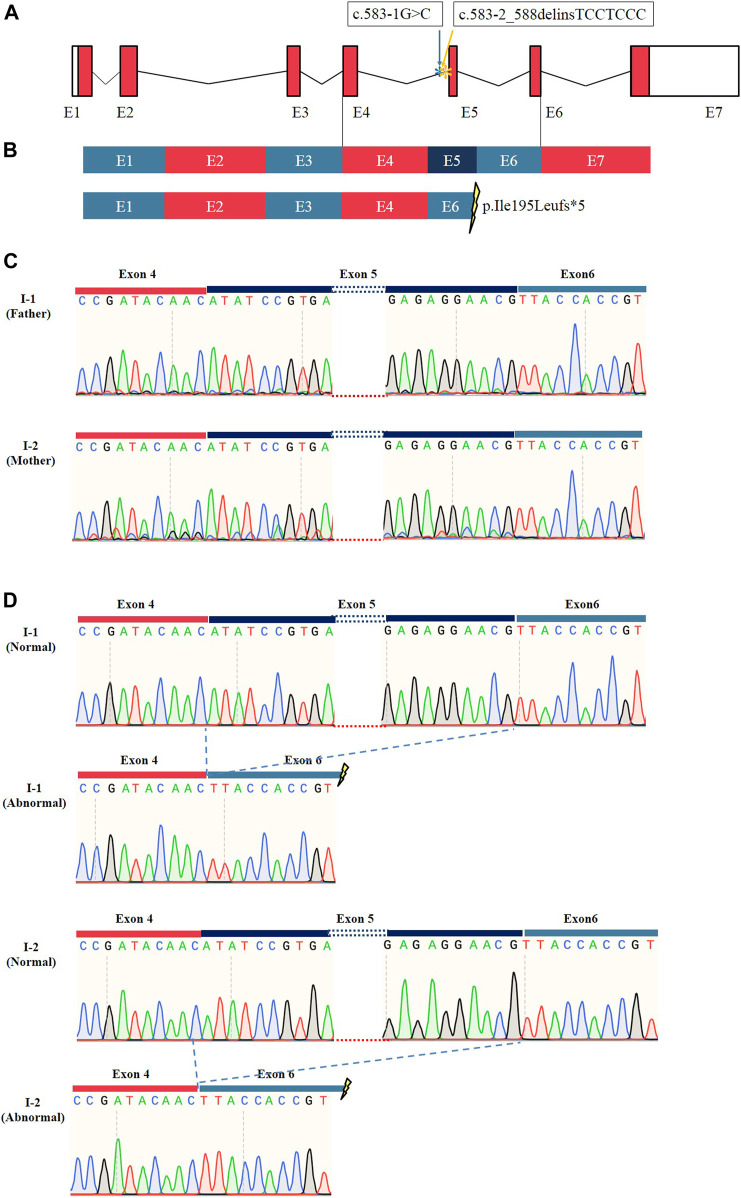
Results of RT-PCR and sequencing of TA clones. **(A)** Schematic representation of the *TMEM231* gene. Asterisks (*) indicate the mutation sites. **(B)** Schematic representation of normal and abnormal post-transcriptional splicing of *TMEM231*. These splice site variants resulted in the loss of exon 5, generating a premature stop codon. E1∼7 indicates exon1∼7. **(C)** Sanger sequencing results of RT-PCR products from I-1 and I-2. **(D)** TA clone sequencing showed that there were two different transcripts of *TMEM231* in the proband’s father (I-1) and mother (I-2).

To determine whether these two splice site mutations affected the post-transcriptional cleavage of *TMEM231*, cDNA from whole blood of the proband’s parents was amplified and sequenced. The results provided strong evidence for alternative transcript variants for both *TMEM231* variants (Figure 3C, Supplementary Figure S3). Accordingly, TA clone and monoclone sequencing was performed. The abnormal transcript was detected and indicated that the two splice site mutations caused the same skipping of the fifth exon of the *TMEM231* gene (Figure 3D). Alternative transcription resulted in an 82 bp frameshift deletion (c.583_664del, p. Ile195Leufs*5) and produced a truncated protein of 198 amino acids (Figures 3A, B).

### These novel splice-site mutations led to decreased mRNA of *TMEM231* and aberrant cilia

**FIGURE 4:**
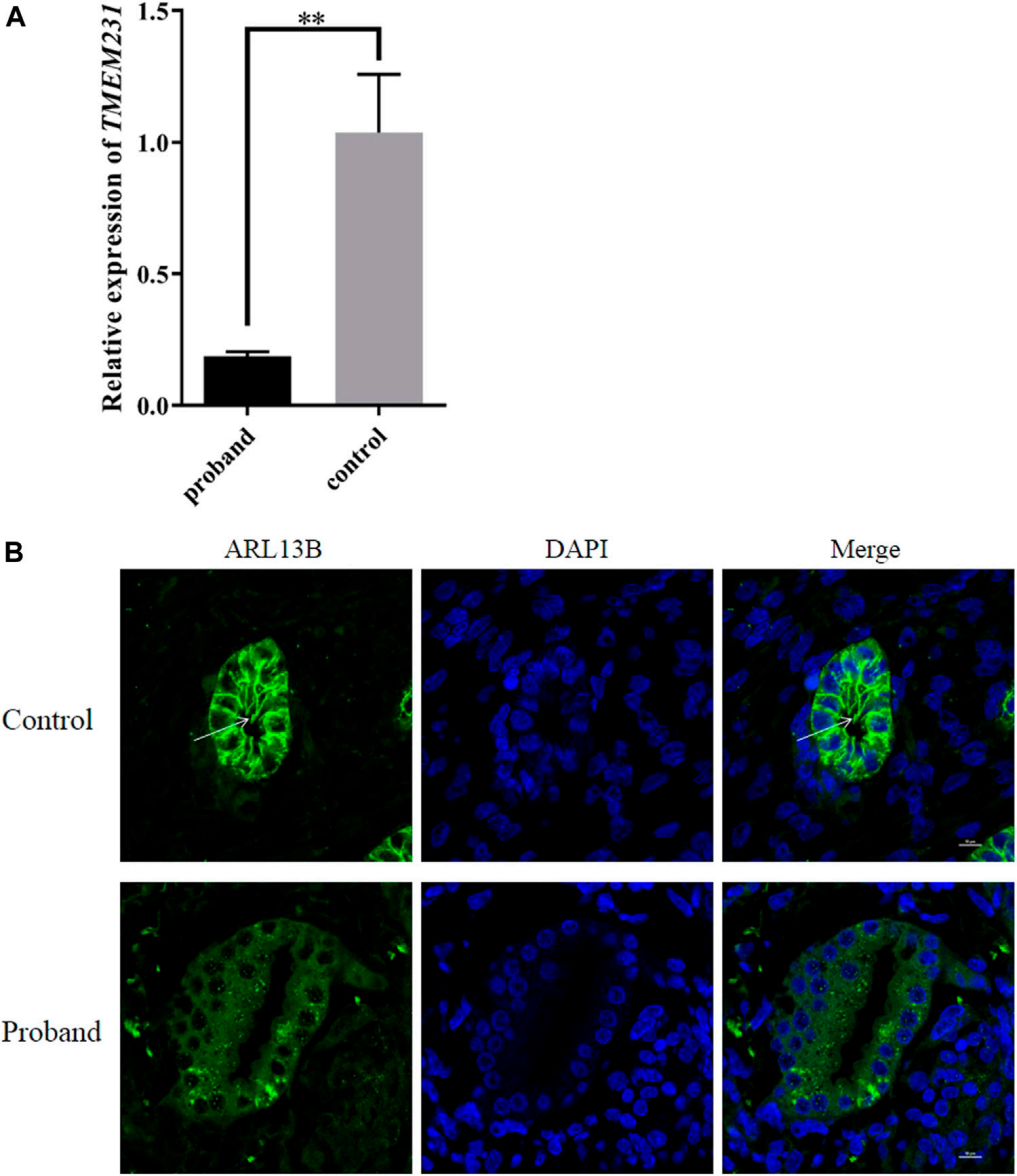
These novel splice site mutations resulted in decreased *TMEM231* mRNA and aberrant cilia. **(A)** Expression of *TMEM231* in the proband’s kidney. Student’s t-test was used for within-sample analysis. Results are expressed as mean ± SEM, **p < 0.01. **(B)** Localisation of ARL13B in affected and control fetal kidney tissue (1,000×). DAPI, for nuclear staining (blue). Alexa-Fluor 488 conjugated secondary antibody was used for ARL13B (green).

To verify whether these novel splice site mutations would cause any change in the expression of *TMEM231 in vivo*, qPCR was performed to detect mRNA levels. The results showed that the expression of *TMEM231* was significantly decreased in tissues from the proband’s kidney, heart and liver (Figure 4A and Supplementary Figure S2).

Primary cilia are specialised sensory organelles that protrude from the surface of most cell types. B9 is a ciliary complex located at the transition zone (TZ) of primary cilia, and TMEM231 constitutes part of the B9 complex. To investigate whether these splice site mutations affect the structures of primary cilia, ADP-ribosylation factor-like protein 13B (ARL13B, a specific marker of primary cilia) was used as the primary antibody in immunofluorescence. We found that the expression of ARL13B was significantly decreased in the kidney of the proband with cystic renal dysplasia, and primary cilia were almost absent in the renal tubular epithelial cell of the proband (Figure 4B, Supplementary Figure S4).

## Discussion

*TMEM231* is a causative gene for MKS. It encodes a 36 KD two-pass transmembrane protein that is part of the B9 complex and localises to the base of the ciliary axoneme at the TZ (Chih et al., 2011). Mutations in mouse *Tmem231* disrupted the ciliary localisation of proteins such as Arl13b and lnpp5e, resulting in the pathogenic features of MKS, and mutations in the *TMEM231* gene were first reported in MKS patients in 2013 (Shaheen et al., 2013; Roberson et al., 2015). In this study, we described the clinical features of a Chinese pedigree with four fetuses affected by MKS, and two novel splice site mutations of *TMEM231* (c.583-1G>C and c.583-2_588delinsTCCTCCC) were identified in this pedigree by WES.

Only 15 *TMEM231*-associated MKS cases were reported, and 16 variants (8 splice sites, 7 missense and 1 small deletion) were considered as causative mutations (Supplementary Table S1) (Roberson et al., 2015; Maglic et al., 2016; Li et al., 2020; Watson et al., 2020). The premature stop-gain mutations caused 80% of the MKS cases (12 out of 15). The c.373C>G (p.Pro125Ala) mutation was detected in four cases of northern Europeans, suggesting that this site may be a mutation hotspot in the northern European population (Roberson et al., 2015). The splice site variant of c.583-1G>A (NM_001077418.2) was found to cause loss of exon 5, generating a premature stop codon (Maglic et al., 2016). Our findings on the c.583-1G>C and c.583-2_588delinsTCCTCCC variants confirmed that mutations within this splice site region lead to the deletion of exon 5 during post-transcriptional cleavage of *TMEM231*. It is speculated that this site may be another mutation hotspot. Actually, the abnormal transcripts were ambiguously observed in the parents (Supplementary Figure S3) and were not detected in the fetus by agarose gel electrophoresis (data not shown). We propose the following hypotheses: (1) a considerable period of time elapsed between the drug-induced abortion and the collection of the fetal sample, during which RNA degradation occurred; (2) the frameshift effect caused by two splicing mutations led to nonsense-mediated mRNA decay (NMD), resulting in a low abundance of RNA that could not be observed in the fetal sample (Amrani et al., 2004; Ivanov et al., 2016).

With the development of next-generation sequencing (NGS), WES has improved the diagnostic rate and increased the knowledge of most disorders (Guo et al., 2022). In particular, WES is the most common approach to identify the candidate gene and disease-causing variants in patients with extreme genotypic and phenotypic heterogeneity disorders. In this study, WES successfully identified disease-causing mutations in the *TMEM231* gene. Due to the poor ability of NGS based on short-read sequencing to accurately detect gene conversion events and distinguish pseudogenes, Watson et al. stated that new third-generation long-read sequencing technologies should be considered as a routine clinical approach for genetic diagnosis (Watson et al., 2020). In their study, a splice site mutation and an exon deletion of the *TMEM231* gene were detected in MKS patients by long-read nanopore sequencing. Therefore, next-generation sequencing and third-generation sequencing could complement each other in clinical diagnosis to improve diagnostic yield.

MKS is a rare, highly clinically and genetically heterogeneous disease. To date, the diagnostic criteria remain controversial. Salonen et al. showed that the minimum diagnostic criteria for MKS include ① polycystic kidneys, ② fibrotic changes of the liver, ③ occipital encephalocele and ④ malformation of the central nervous system (Salonen, 1984). All 15 reported MKS patients had abnormal fetal features and had a poor prognosis. Cystic dysplasia was a common manifestation in all patients. Hepatic developmental defects were also a common phenotype, but were not manifested in the fetuses in this study. (Supplementary Table S1). However, liver fibrosis and bile duct proliferation have been reported to occur in only 65.5% of MKS cases (Barisic et al., 2015). In this study, we described a pedigree with four fetuses affected by MKS. The proband (Ⅱ-4) not only presented the typical features of MKS, but also showed bilateral talipes equinovarus. Ⅱ-3 presented compatible with typical features of MKS, whereas Ⅱ-1 and Ⅱ-2 did not show polydactyly. In addition, the proband (Ⅱ-4) did not have any liver developmental defects at autopsy. MKS also shares clinical features with several cilia-based disorders such as JTBS and BBS. Due to the complexity of MKS phenotypes, accurate diagnosis of MKS is usually difficult and delayed. Therefore, appropriate ancillary examinations and genetic testing are required.

Genotype-phenotype correlations are essential for clinical research. In this study, these two splice site mutations caused different phenotypes in four affected fetuses, and we hypothesised that these mutations would lead to nonsense-mediated mRNA decay (NMD). Although the pathogenesis of MKS caused by mutations in *TMEM231* remains unclear, we have shown that the defective TMEM231 protein results in a developmental defect in primary cilia (Figure 4, Supplementary Figure S4). Accurate splice site recognition is critical for pre-mRNA splicing. RT-PCR followed by Sanger sequencing showed that these two splice site mutations altered the post-transcriptional cleavage of *TMEM231*. TA cloning sequencing revealed the same alternative transcription in this nonconsanguineous couple. Some researchers have used RNA-seq to explore novel splice sites. On the other hand, RNA-seq can also be used to determine the impact of splicing variants on the transcriptome (Parada et al., 2014). For example, Zhi et al. reported two aberrant transcripts caused by a c.3353 + 5C>A variant of *MED12* by RNA-seq (Zhi et al., 2022). Multiple aberrant transcripts caused by transcript splicing may be the reason for the negative genotype-phenotype association in genetic counselling. Although RNA-seq is powerful for detecting different transcripts, the specificity of gene expression in different tissues should be considered before performing RNA-seq.

In conclusion, we reported two novel splice site mutations (c.583-1G>C and c.583-2_588delinsTCCTCCC) of *TMEM231* in a Chinese pedigree with four affected fetuses. Considering the clinical phenotype of the pedigree and the functional analysis of two mutations, we believed that all fetuses were affected with MKS and that the mutations were pathogenic mutations causing MKS. The results of RT-PCR and TA clone sequencing showed alternative post-transcriptional cleavage of *TMEM231*, resulting in a deletion of exon 5 and a premature stop codon in *TMEM231* mRNA. The absence of primary cilia in renal tubular epithelial cells partially explained the mechanism by which *TMEM231* gene mutations affect primary cilia development and formation. This study also provided a basis for genetic counselling of this pedigree and extended the mutation spectrum of the *TMEM231* gene for MKS.

## Data Availability

The variation data reported in this paper has been deposited in the Genome Variation Map in National Genomics Data Center, China National Center for Bioinformation/Beijing Institute of Genomics, Chinese Academy of Sciences, under accession number GVM000604 that can be publicly accessible at http://bigd.big.ac.cn/gvm/getProjectDetail?project=GVM000604.
