# Supplementary figures and images for: Identification of novel TMEM231 gene splice variants and pathological findings in a fetus with Meckel Syndrome
Source: Front Genet. 2023 Sep 6;14:1252873. doi: 10.3389/fgene.2023.1252873 (PMC10509762; doi:10.3389/fgene.2023.1252873)

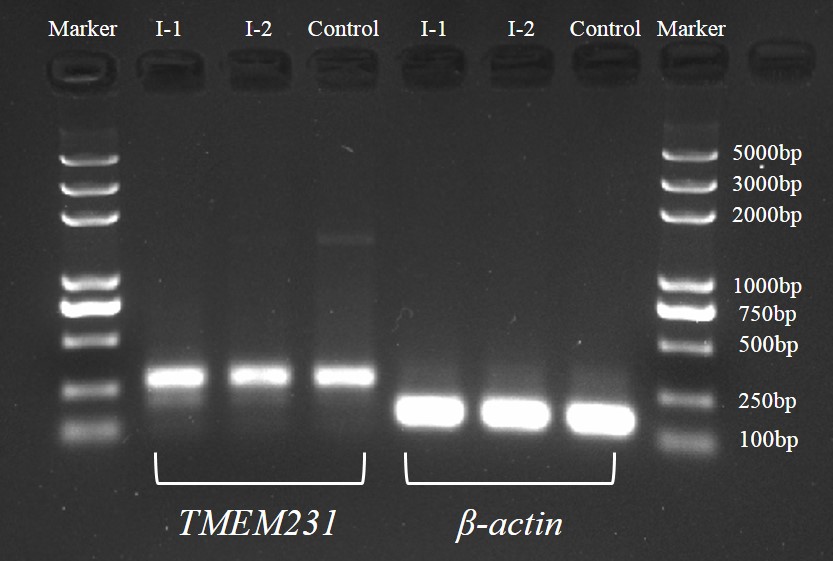

Supplement: Supplementary file 1 [file Image3.JPEG]

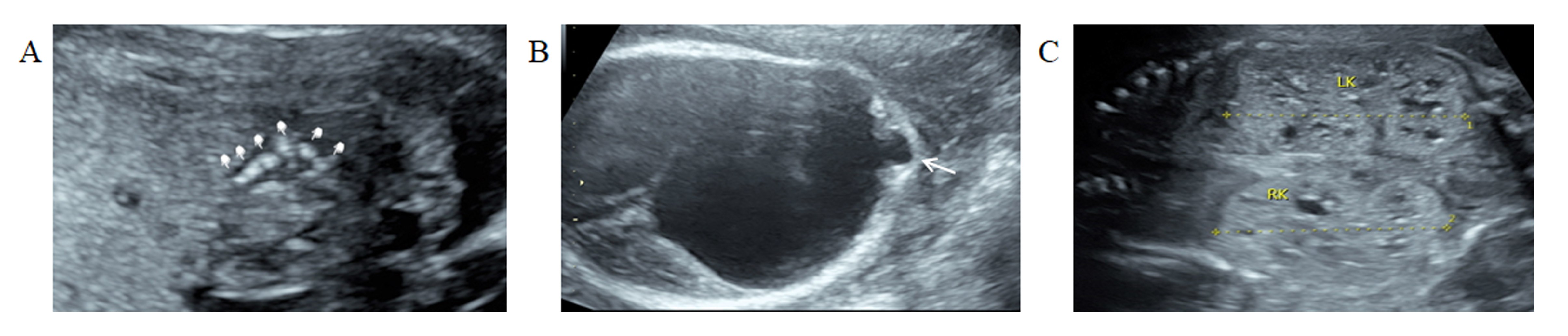

Supplement: Supplementary file 2 [file Image1.JPEG]

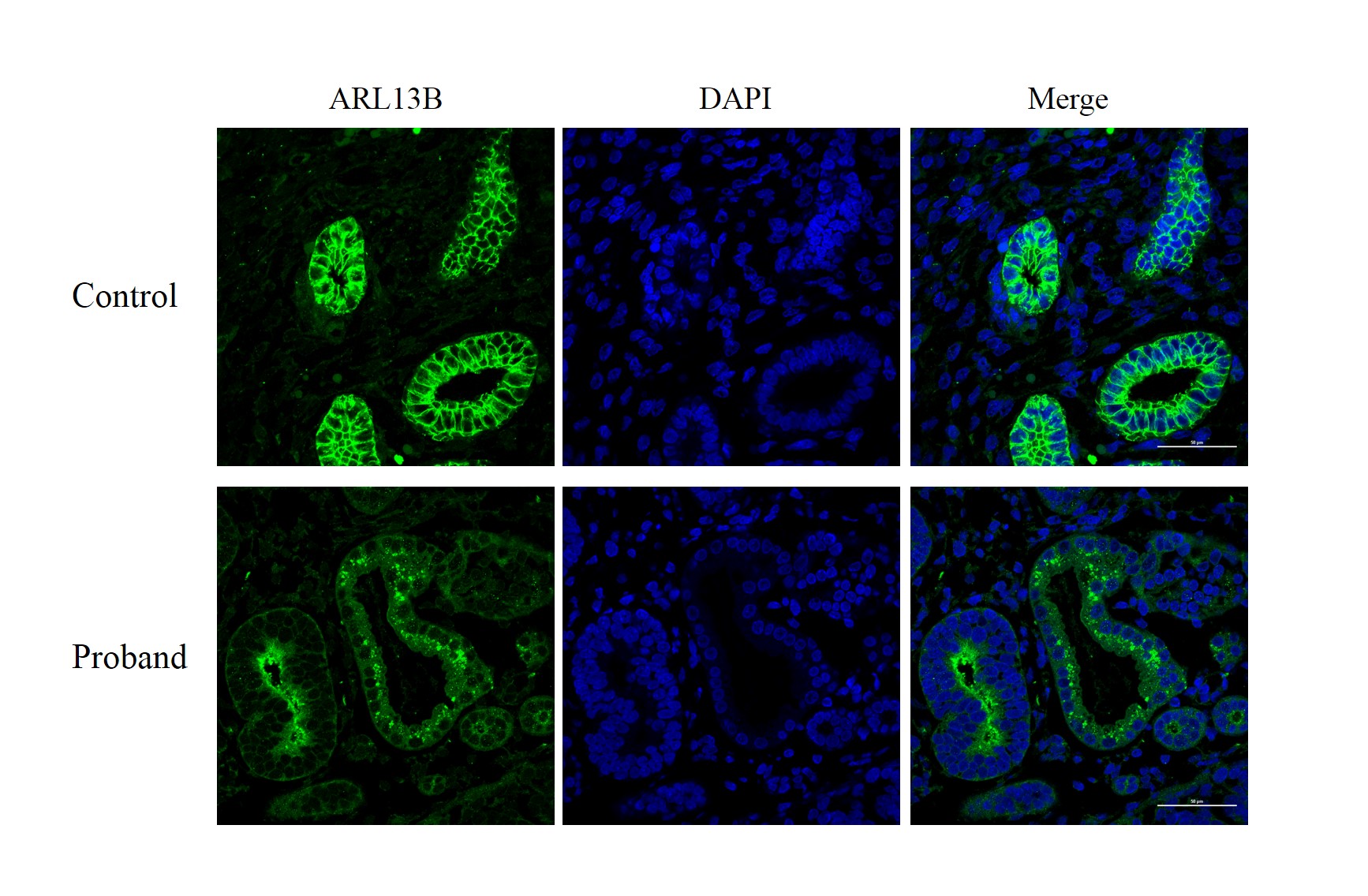

Supplement: Supplementary file 3 [file Image4.JPEG]

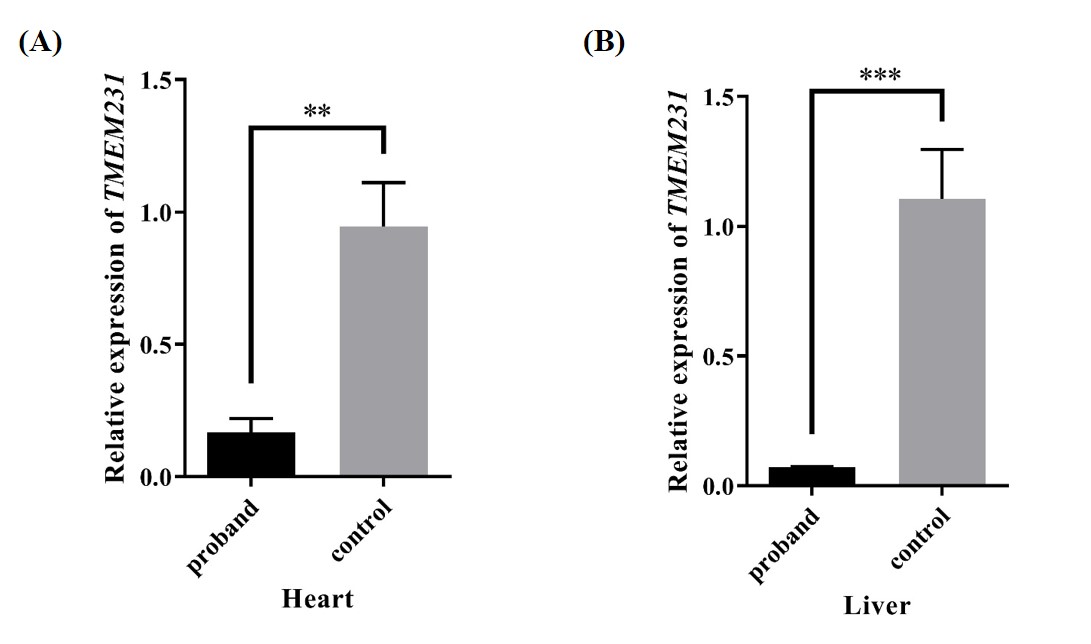

Supplement: Supplementary file 4 [file Image2.JPEG]
