# Supplementary material for: Identification of novel TMEM231 gene splice variants and pathological findings in a fetus with Meckel Syndrome
Source: Front Genet. 2023 Sep 6;14:1252873. doi: 10.3389/fgene.2023.1252873 (PMC10509762; doi:10.3389/fgene.2023.1252873)
Supplement: Supplementary file 5 [file Table2.DOCX]

Supplementary Material

Identification of novel *TMEM231* gene splice variants and pathological findings in a fetus with Meckel Syndrome

Qian Zhang^1,2†^, Shuya Yang^3†^, Xin Chen^1,2^, Hongdan Wang^1,2^, Keyan Li^2^, Chaonan Zhang^2^, Shixiu Liao^1,2^, Litao Qin^1,2*^, Qiaofang Hou^1,2,3,*^

*** Correspondence:** Qiaofang Hou, houqf_2000@163.com; Litao Qin, [litao_qin@zzu.edu.cn](mailto:litao_qin@zzu.edu.cn)

# Supplementary Figure


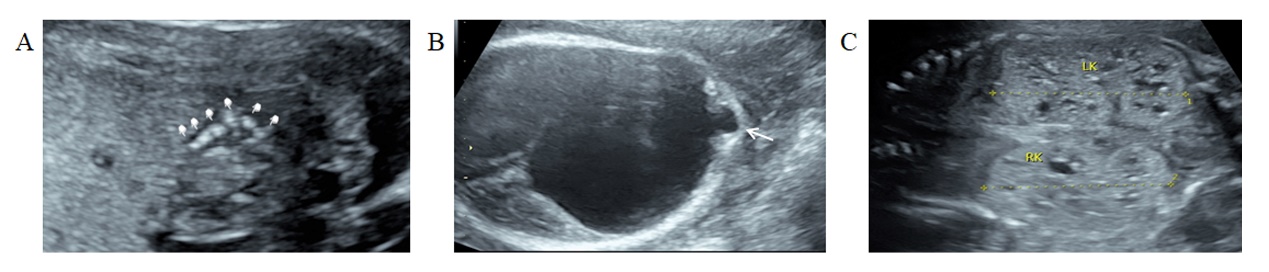


**Supplementary Figure 1.** Clinical features of the third fetus (Ⅱ-3). (A) (B), and (C) The ultrasound examination revealed postaxial polydactyly, occipital encephalocele, and bilateral polycystic kidney.


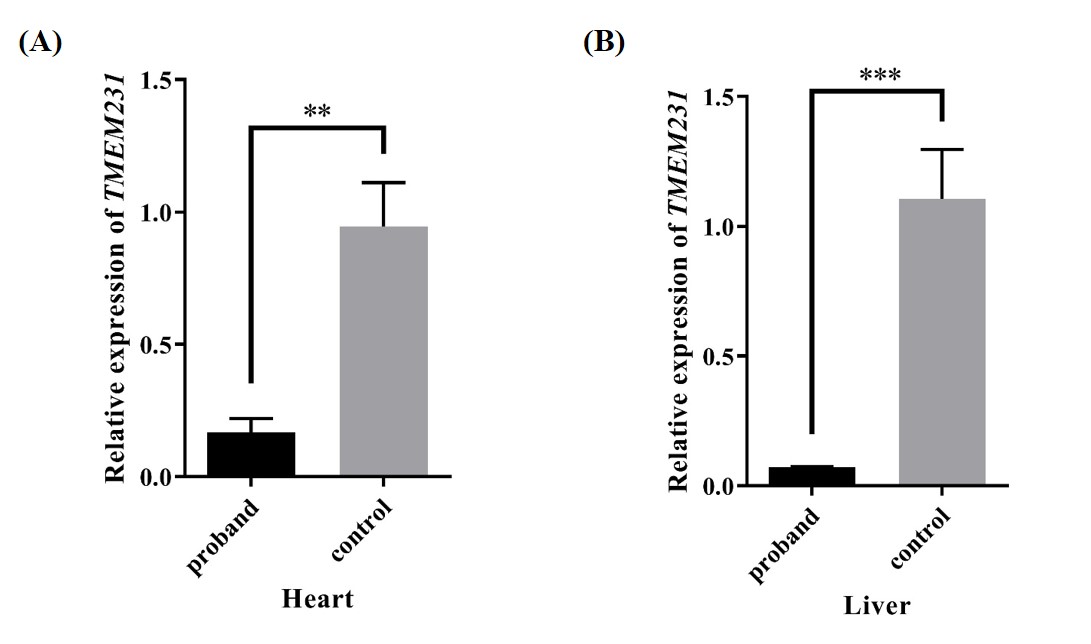


**Supplementary Figure 2.** (A) Expression of *TMEM231* in the heart of the proband. (B) Expression of *TMEM231* in the liver of the proband. Student's t-tests were used for within-sample analyses. Results are presented as mean ± SEM, ^**^P<0.01, ^***^P<0.001.


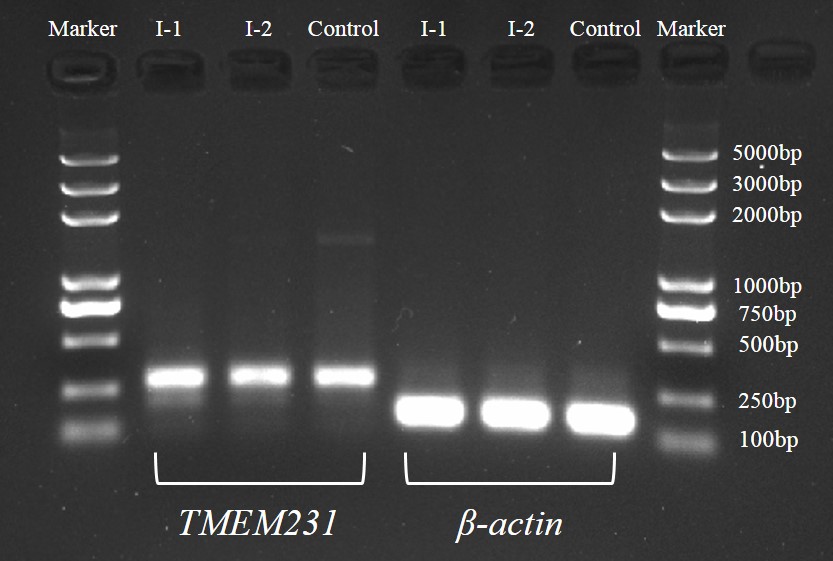


**Supplementary Figure 3.** Agarose gel electrophoresis for PCR amplification products of parental cDNA. The primers used are listed in Table 1 (Primer #2 and Primer #5).


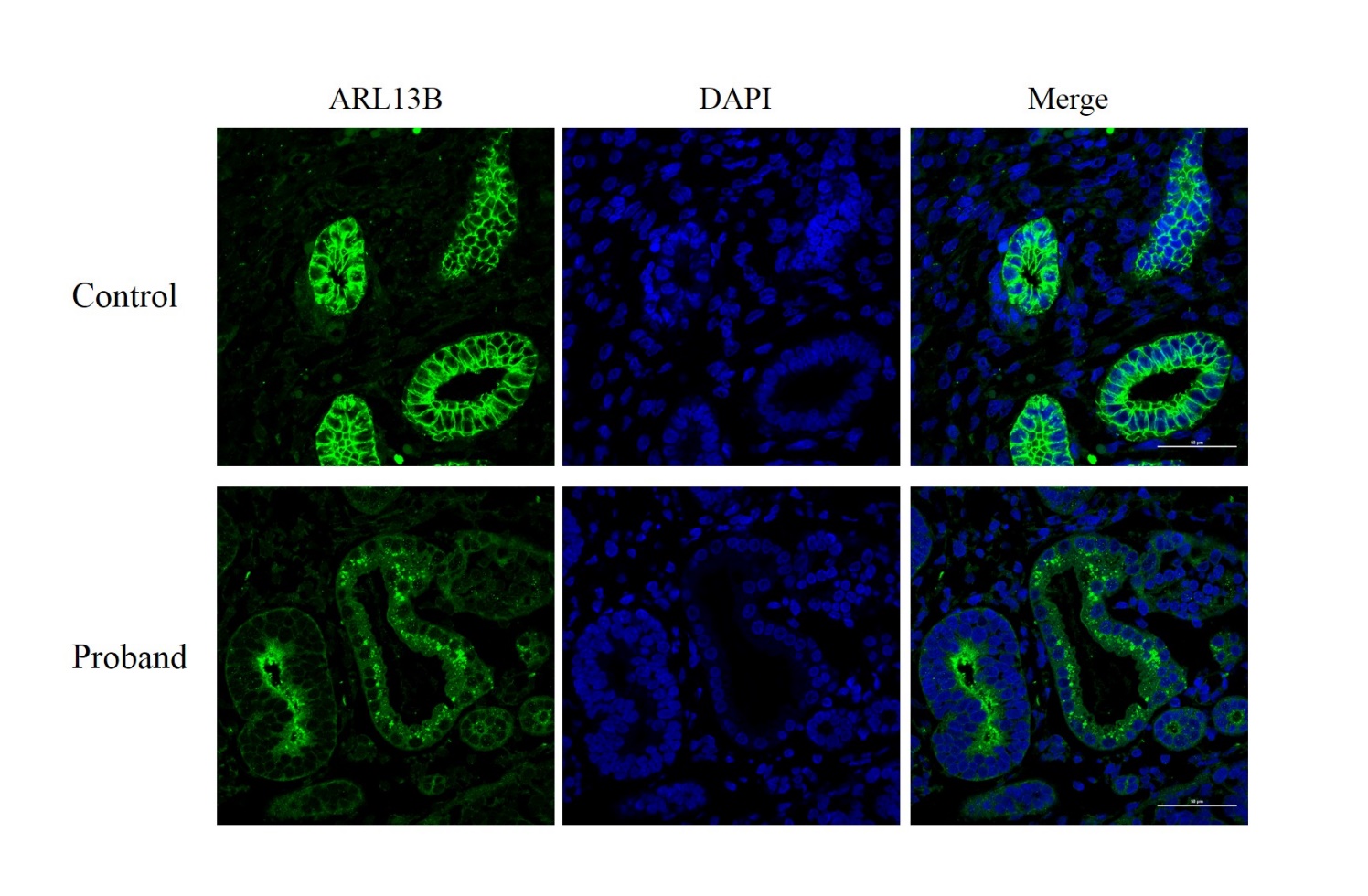


**Supplementary Figure 4.** Immnofluorescence figure with a larger field of view to reflect the overall situation. Localisation of ARL13B in affected fetal and control fetal kidney tissues (600×). DAPI, for nuclear staining (blue). Alexa-Fluor 488 conjugated secondary antibody was used for ARL13B (green).
